# Biogenic silica accumulation in picoeukaryotes: Novel players in the marine silica cycle

**DOI:** 10.1111/1758-2229.13144

**Published:** 2023-03-29

**Authors:** Yelena Churakova, Anabella Aguilera, Evangelia Charalampous, Daniel J. Conley, Daniel Lundin, Jarone Pinhassi, Hanna Farnelid

**Affiliations:** ^1^ Centre for Ecology and Evolution in Microbial Model Systems (EEMiS) Linnaeus University Kalmar Sweden; ^2^ Department of Geology Lund Sweden

## Abstract

It is well known that the biological control of oceanic silica cycling is dominated by diatoms, with sponges and radiolarians playing additional roles. Recent studies have revealed that some smaller marine organisms (e.g. the picocyanobacterium *Synechococcus*) also take up silicic acid (dissolved silica, dSi) and accumulate silica, despite not exhibiting silicon dependent cellular structures. Here, we show biogenic silica (bSi) accumulation in five strains of picoeukaryotes (<2–3 μm), including three novel isolates from the Baltic Sea, and two marine species (*Ostreococcus tauri* and *Micromonas commoda*), in cultures grown with added dSi (100 μM). Average bSi accumulation in these novel biosilicifiers was between 30 and 92 amol Si cell^−1^. Growth rate and cell size of the picoeukaryotes were not affected by dSi addition. Still, the purpose of bSi accumulation in these smaller eukaryotic organisms lacking silicon dependent structures remains unclear. In line with the increasing recognition of picoeukaryotes in biogeochemical cycling, our findings suggest that they can also play a significant role in silica cycling.

## INTRODUCTION

Annually, oceans receive an input of nearly 15 Tmol silicon (Tréguer et al., 2021) in the form of silicic acid (dissolved silica, dSi), which is biologically integrated and transformed into amorphous silica by organisms known as biosilicifiers. Diatoms are thought to be the dominant biosilicifying phytoplankton group, making carbon cycling coupled with silica cycling and other biogeochemical cycles, because they in large part enable primary production and subsequent exports of carbon and silica into deep oceans (Tréguer & De La Rocha, 2013; Tréguer & Pondaven, 2000). The silicified cell walls facilitate the sinking of diatoms from surface waters into deep ocean sediments (Smetacek et al., 2012; Yool & Tyrrell, 2003), making them important carbon exporters. In diatoms, dSi uptake is directly tied to the cell cycle and silicon, like other macronutrients such as carbon, nitrogen and phosphorus, is necessary for growth and metabolism (Claquin & Martin‐Jézéquel, 2005; Hildebrand, 2000; Martin‐Jézéquel et al., 2000). These findings emphasize the important linkages between silicon and other nutrients for regulating ocean productivity and biogeochemical cycles.

Until recently, primary control over biological silica cycling in the oceans was largely attributed to diatoms due to their effective silicon ion transporters (SITs), which are used to actively take up dSi (Hildebrand et al., 1997, Thamatrakoln & Hildebrand, 2008). It was generally assumed that microorganisms lacking SITs and silicon dependent cellular structures were not able to take up dSi, especially at the low concentrations found at the surfaces of modern oceans, which have trended significantly downwards from those found in ancient oceans (Conley et al., 2017). Recent discoveries, however, are beginning to change this paradigm, and earlier studies exploring the utilization of dSi by non‐silicifying nanoplankton have been validated by recent research efforts (Fisher et al., 1991; Nelson et al., 1984). We now know that SITs or SIT‐like (SIT‐L) transporters are not exclusive to diatoms (Durak et al., 2016; Marron et al., 2016), nor even necessary for biogenic silica (bSi) accumulation (Tostevin et al., 2021), and that bSi can be produced by non‐silicified organisms with and without an obligate need for dSi. Still, our understanding of silica utilization in these organism groups is limited.

Field, cultivation and molecular studies have recently uncovered biosilicifiers among taxa that are traditionally not considered to utilize silica, both with and without genes encoding SITs. Notably, a study of unicellular cyanobacteria belonging to the genus *Synechococcus* from the Sargasso Sea and eastern equatorial Pacific, revealed substantial bSi accumulation (Baines et al., 2012). This was remarkable as, thus far, in silico analysis have revealed SIT‐L sequences only in two *Synechococcus* isolates (*Synechococcus* sp CC9616 and KORDI‐100) (Marron et al., 2016). Laboratory studies of *Synechococcus* strains without SIT‐L genes confirm that cellular silicon accumulation increases with higher concentrations of dSi in the media (Brzezinski et al., 2017; Tostevin et al., 2021). However, so far, no differences in growth rates between cells taking up dSi and control cultures have been observed and no role or benefit of taking up dSi is documented (Brzezinski et al., 2017, Tostevin et al., 2021). It is noteworthy that although the bulk alkaline based digestion method is still widely utilized to analyse bSi, it does not directly measure bSi, but cellular Si. Averaging field and model estimates, indicate a marine gross bSi production at 255 (±52) Tmol silicon yr^−1^, of which up to 7.9% is potentially attributed to picocyanobacteria (Tréguer et al., 2021). A recent study calculated that picophytoplankton (<2–3 μm in diameter), including picocyanobacteria and picoeukaryotes, contribute to bSi standing stocks and production, as well as overall flux, in much more significant proportions (Wei & Sun, 2022). Estimates include a bSi production rate of 32–80% of 240 Tmol Si yr^−1^, and responsibility for approximately 55% of global annual ocean Si flux. In parallel with these discoveries, some recent field studies have shown that pico‐sized phytoplankton also accumulate significant amounts of bSi in various oligotrophic ocean areas like the North Atlantic, Sargasso Sea, South Pacific, North Pacific, Eastern Indian Ocean and Western Pacific Ocean (Krause et al., 2017; Leblanc et al., 2018; Ohnemus et al., 2016; Wei, Sun, et al., 2021; Wei, Wang, et al., 2021; Wei, Zhang, et al., 2021). The results of these field studies warrant confirmation and more in‐depth investigations of bSi production by picophytoplankton in field and laboratory settings.

Picophytoplankton are highly abundant and distributed globally in modern oceans (Biller et al., 2015; Worden & Not, 2008) and future projected warming conditions and increased stratification are expected to promote a significant increase of their total global biomass (Flombaum et al., 2013; Flombaum et al., 2020; Iudicone, 2020). Picoeukaryotes greatly contribute to phytoplankton biomass, productivity and diversity—especially in coastal waters (Buitenhuis et al., 2012; Li, 1994; Not et al., 2005; Vaulot et al., 2008; Worden & Not, 2008). They have primarily been characterized through 18S rRNA gene sequencing and flow cytometry studies (Jardillier et al., 2010; Simon et al., 1994; Worden et al., 2004). The contribution of picoeukaryotes to primary productivity is considerably higher than their cell abundances suggest (Li, 1994; Worden et al., 2004), and they are significant players in oceanic carbon cycling (Rii et al., 2016). Picoeukaryotes have been historically understudied in comparison to picocyanobacteria, and their importance to oceanic ecosystems continues to be evaluated. The taxonomic classification of picoeukaryotes is still a work in progress, but cultured representatives of major lineages are available (Guillou et al., 2004). *Ostreococcus tauri* and *Micromonas commoda* are representative strains of the two families within Mamiellales, an order of green algae that includes some of the more abundant and well‐studied marine picoeukaryotes (Demir‐Hilton et al., 2011; Vaulot et al., 2008; Worden et al., 2009). Though the comprehensive view of the biogeographical distribution of Mamiellales is still limited, due to a lack of reference genomes, various studies have affirmed their relevance in coastal surface waters (Demir‐Hilton et al., 2011; Worden et al., 2004). Despite their present and future importance to nutrient cycling, their contribution to oceanic silica cycling is still unexplored.

To investigate novel biosilicifiers, we carried out laboratory experiments to study bSi accumulation in five picoeukaryote isolates that seemingly have no obligate need for silica. Three picoeukaryote strains, isolated from seawater collected from the coastal Baltic Sea, as well as two model marine picoeukaryote strains, *O. tauri* and *M. commoda*, were the focus of our experiments. Plastid 16S rRNA and 18S rRNA genes of the novel isolates were sequenced and published datasets were screened to determine the relative abundance of the strains in the Baltic Sea. This study is the first to document accumulation of bSi by picoeukaryotes lacking silicon dependent structures. The results of this investigation offer novel insights into silicon uptake in picoeukaryotes and suggest that coastal picoeukaryotes may have a significant role in oceanic silica cycling.

## EXPERIMENTAL PROCEDURES

### Environmental sampling and isolation of strains

Water samples were collected at a coastal station in Kalmar, Sweden in the Baltic Sea, K‐station (56°39'25.4"N and 16°21'36.6" E, 1 m sampling depth). Temperature and salinity were measured with a CTD Castaway conductivity/temperature/depth sensor. Samples were initially filtered through a 200 μm mesh to remove large particles. For strain isolation, water aliquots were pre‐filtered through a 3 μm polycarbonate filter, adjusted with supplementary nitrate NO_3_ (580 μM) and phosphate PO_4_ (56.6 μM), and incubated at 16, 18 or 20°C at an irradiance of 15 μmol m^−2^ s^−1^ with a light: dark cycle of 12:12 h. After 24 h of incubation, the isolates were serially diluted in 24‐well plates with L1 media (Guillard & Hargraves, 1993), prepared using Baltic seawater with a salinity of approximately 7 PSU, NO_3_ (882 μM), and PO_4_ (36.2 μM). When colour was visible in individual wells, cells were transferred to 40 ml plastic culture flasks and grown in L1 media prepared with artificial sea water (7 PSU). The morphology and purity of the isolates, defined as only one visual autotrophic morphotype, were examined using an epifluorescence microscope (Olympus BX50) at ×1000 magnification.

### Molecular identification of Kalmar algae collection (KAC) strains

For DNA extraction, 4 ml of culture was centrifuged for 8 min at 8000×*g* to form a cell pellet, which was stored at −80°C until extraction. The FastDNA SPIN Kit for Soil (MP Biomedicals) was used according to the manufacturer's instructions to extract DNA with Matrix E columns and the addition of proteinase K (1% final concentration). Samples were incubated at 55°C for 1 h directly after homogenization. The concentration of extracted DNA was measured using an Invitrogen Qubit 2.0 Fluorometer (Thermo Fisher Scientific) and its purity was assessed with a NanoDrop 2000 Spectrophotometer (Thermo Fisher Scientific). Universal primers 27F and 1492R were used to amplify plastid 16S rRNA (Lane, 1991). Primers Reuk454FWD1 and 981 V4r covering the V4‐V5 region were used to amplify 18S rRNA (Bradley et al., 2016; Stoeck et al., 2010). The sequences were amplified in a final volume of 50 μl including 10 ng of DNA template, 0.5 μM of each primer, and 2× Phusion High‐Fidelity PCR Master Mix (Thermo Fisher Scientific) on a T100 Thermal Cycler (Bio‐Rad Laboratories, USA). The amplification reactions were run with the following cycling conditions: initial denaturation at 98°C for 30 s, followed by 20 cycles at 98°C for 10 s, annealing at 55°C for 30 s for 16S rRNA or 57°C for 45 s for 18S rRNA, extension at 72°C for 15 s and a final extension at 72°C for 2 min. The amplified products were sent for Sanger sequencing (Macrogen Europe, Netherlands). All sequences generated in this study were submitted to GenBank under accession numbers ON969163–ON969165 and ON951866–ON951868 for the 16S and 18S rRNA sequences, respectively. The 16S rRNA gene amplicon sequence library from the Linnaeus Microbial Observatory (LMO) (2011–2020) was used to investigate the relative abundance of amplicon sequence variants (ASVs) with 100% identity to KAC isolates. The data is available at the European Nucleotide Archive (ENA) under accession numbers PRJEB52627, PRJEB52772, PRJEB52496, PRJEB52780, PRJEB52782, PRJEB52828, PRJEB52837, PRJEB52851 and PRJEB52854.

### Culture conditions and set up for bSi accumulation experiments

Prior to the experiments, all cultures were acclimatized for, at minimum, 1 month in L1 media without added dSi. During the experiments, three selected KAC isolates (KAC117, KAC118 and KAC119), plus *Ostreococcus tauri* (RCC4221) and *Micromonas commoda* (RCC827) strains purchased from the Roscoff Culture Collection (RCC; Roscoff, France) were grown in L1 media prepared with 7 and 33 PSU artificial seawater, respectively, in polycarbonate bottles at 18°C, at 100 μmol photons m^−2^ s^−1^ irradiance on a 12 h light:12 h dark photocycle. The picoeukaryotes were cultured in media with and without added dSi (100 μM). pH was <8.5 throughout the experiments (Figure S3, see Discussion) and was controlled by bubbling with ambient air, which was sterilized by passage through a 0.2 μm pore‐size Nuclepore Track‐Etch filter (Whatman, UK) prior to entering each culture bottle.

Culture growth was monitored by measuring the optical density approximately every 24 h at a wavelength of 750 nm using a FLUOstar Omega Microplate Reader (BMG Labtech, Germany). 20–30 ml of exponentially growing cells from each culture were filtered onto 47 mm diameter 0.2 μm pore‐size Nuclepore Track‐Etch filters (Whatman, UK) to determine biogenic silica quotas, which were tested as a function of dSi concentration. The filters were stored in Teflon tubes at −20°C until digestion in 0.2 M NaOH at 95°C for 1 h following Krause et al. (2013) and Brzezinski et al. (2017). Biogenic silica was transformed into dSi and measured with a UV‐1600PC Spectrophotometer (Shimadzu, Japan) at a wavelength of 810 nm using 50 mm cuvettes. Cell suspensions (5 ml) were filtered through a 33 mm diameter 0.22 μm pore‐size Millex‐GP filter (Millipore, Ireland) and stored at −20°C to determine the concentration of dSi following Hansen and Koroleff (1999). Aliquots (1 ml) were fixed with Lugol's solution for cell counts and size measurements using a microscope (Olympus BX50) at ×1000 magnification. Cell size measurements were estimated by uploading images onto Adobe Photoshop version 22.3.0 and using the ruler tool to average the diameters of 30 cells. Cell sizes (μm) were used to estimate the average biovolume (μm^3^) using geometric shapes (sphere and spheroid) (Hillebrand et al., 1999; Olenina et al., 2006; Sun & Liu, 2003).

### Measurement of dSi concentration in media

To measure the background concentration of dSi in the media, samples were collected at the final timepoints of the experiments. The high salinity media (33 PSU) had higher levels of average dSi (average 2.99 μM) compared with the low salinity (7 PSU) media (average 0.70 μM). This is likely explained by the naturally occurring higher concentrations of dSi in the 33 PSU L1 media, which is connected to the higher levels of sea salt (and other minerals) in the 33 PSU L1 media.

### Data analysis

All experiments were conducted in biological triplicates, and the results are presented as the mean value (±SD). Three independent experiments (E1, E2 and E3) were performed with the KAC isolates and one with the RCC strains. Specific growth rates were calculated using cell abundances (CA) in the exponential phase following the equation:

$$\mu =\frac{\ln\left(\frac{{\mathrm{CA}}_{t_{f}}}{{\mathrm{CA}}_{t_{i}}}\right)}{t_{f}-t_{i}}$$

where *t*_*f*_ and *t*_*i*_ represent the final and initial days of the exponential phase, respectively. Mean and SD were calculated and independent *t* tests were performed in GraphPad Prism version 9.3.1 for MacOS (GraphPad Software, San Diego, CA, www.graphpad.com). ASV relative abundance figures were made in R version 1.4.1106 (R Core Team, 2021) using the package ggplot2 (Wickham, 2016).

## RESULTS

### Characterization of picoeukaryotes used in bSi accumulation experiments

Analysis of the 16S rRNA and 18S rRNA genes from the three novel Kalmar Algae Collection (KAC) isolates showed that they were members of the class Trebouxiophyceae (Table 1). KAC 117 had 16S and 18S rRNA sequences identical to two different *Choricystis* species isolated from freshwater environments. KAC 118 and KAC 119 18S rRNA sequences matched to the same *Nannochloris* species, while their 16S rRNA sequences were similar to two different *Picochlorum* species, indicating that the two isolates are different strains (Table 1). 16S rRNA ASVs identical to the isolate sequences were found in the multiyear Linnaeus Microbial Observatory (LMO) amplicon sequence library (2011–2020). An ASV identical to KAC 117 was detectable throughout different seasons and years. In contrast, the ASV associated with KAC 118 and KAC 119 was detected more sporadically (Figure S1).

**TABLE 1:** List of isolates used in this study, and closest relatives found using NCBI Blastn

Isolate	Isolation date (YYYY‐DD‐MM)	Strain origin	Cell shape	Average biovolume (μm^3^)	Closest relative (16S rRNA); accession no.	Identity (%)	Closest relative (18S rRNA); accession no.	Identity (%)	References
KAC 117	2019‐16‐04	Baltic Sea, brackish	Sphere	2.03	*Choricystis* sp. ACT 0607; MK397009	100	*Choricystis limnetica*; MT423986	100	This study
KAC 118	2019‐02‐04	Baltic Sea, brackish	Sphere	1.26	*Picochlorum* sp.; MN647759	97.63	*Nannochloris* sp.; LC189144	100	This study
KAC 119	2019‐14‐05	Baltic Sea, brackish	Sphere	1.29	*Picochlorum* sp.; MG552671	95.31	*Nannochloris* sp.; LC1891441	100	This study
RCC 827	1998‐10‐02	Pacific Ocean, marine	Sphere	3.99	–	–	*Micromonas commoda*	–	van Baren et al. (2016)
RCC 4221	1995‐03‐05	Mediterranean Sea, marine	Spheroid	1.32	–	–	*Ostreococcus tauri*	–	Chrétiennot‐Dinet et al. (1995)

*Note*: RCC strain information including isolation date, growth temperature and strain identification was retrieved from the RCC website (www.roscoff‐culture‐collection.org). The average biovolume was calculated using cell diameters measured during exponential phase.

**FIGURE 1:**
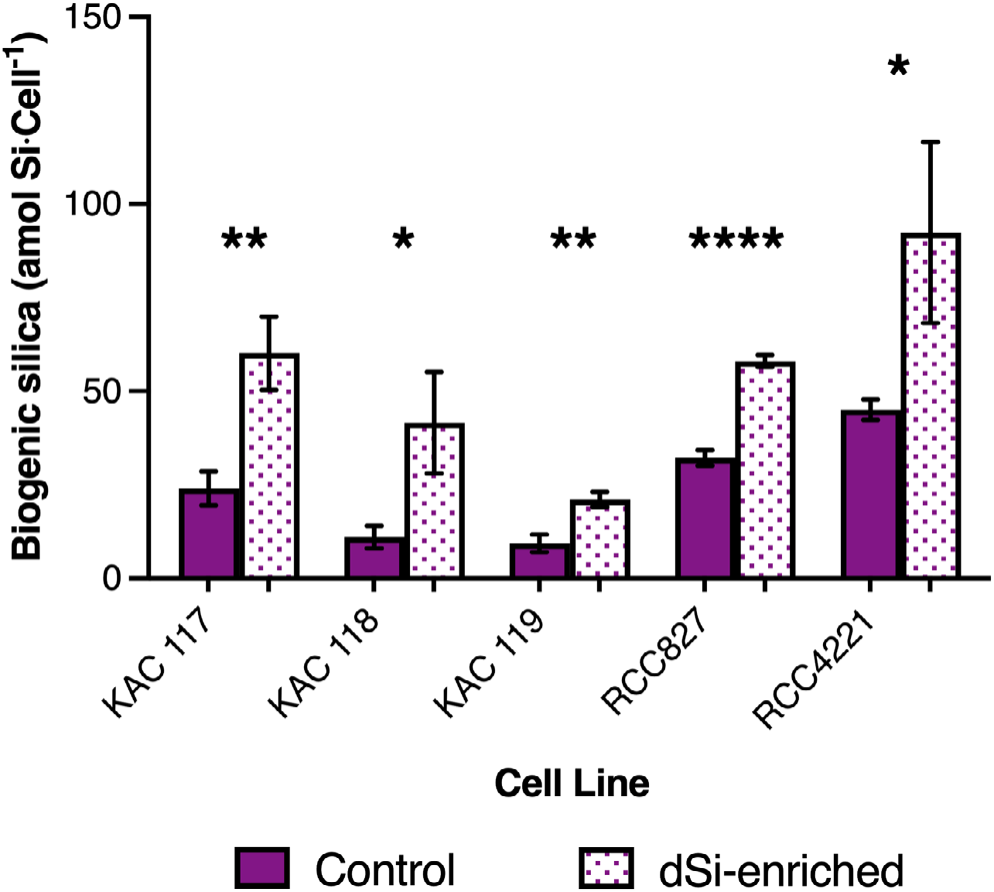
Biogenic silica accumulation in Kalmar Algae Collection (KAC) picoeukaryote strains (KAC 117, KAC 118 and KAC 119) and *M. commoda* strain RCC827 and *O. tauri* strain RCC4221. Strains were cultured in control conditions (without added dSi) and in dSi‐enriched media (+100 μM). The plot shows the results of a single representative experiment (E1). Results from all experiments are shown in Table S1. Asterisk(s) (*) indicate significant *p* values (independent *t* test, * *p* ≤ 0.05, ** p ≤ 0.01, *** p ≤ 0.001, **** p ≤ 0.0001).

All picoeukaryote strains accumulated higher levels of bSi when cultured in media with added dSi, compared with control conditions (Figure 1). To evaluate their capacity to accumulate bSi, we carried out three separate experiments with the KAC isolates. The bSi accumulated in dSi‐enriched cultures was consistently higher in all strains compared with the controls (Table S1). KAC 117 had the highest average values of bSi in both control conditions (30.87 ± 6.58 amol Si cell^−1^) and dSi‐enriched media (62.09 ± 16 amol Si cell^−1^; Table S1). KAC 118 average bSi in control (16.18 ± 6.14 amol Si cell^−1^) and dSi‐enriched (44.66 ± 14.43 amol Si cell^−1^) cultures ranged in between the values measured for KAC 117 and KAC 119 and, though they varied between experiments, the differences in accumulation between control and dSi‐enriched cultures were significant (Table S2). KAC 119 had the lowest average values of bSi in control conditions (12.1 ± 4.95 amol Si cell^−^1) and in dSi‐enriched media (30.31 ± 11.09 amol Si cell^−1^; Table S1). Marine strains RCC827 and RCC4221 had average bSi values similar to KAC 117 in control conditions (32.15 ± 2.07, 45.05 ± 2.76 amol Si cell^−1^, respectively). The average bSi accumulation for RCC827 grown in dSi‐enriched media was most similar to KAC 117 (58.04 ± 1.62 amol Si cell^−1^). Overall, RCC4221 accumulated the highest average cellular silicon in media with added dSi, measuring 92.41 ± 24.14 amol Si cell^−1^. Our data does not support a relationship between cell size or volume of the different strains and cellular accumulation (Table S3). The growth and pH curves of the picoeukaryotes were similar when grown in control and dSi‐enriched cultures (Figures S2 and S3) and mean growth rates (0.93–1.83 Day^−1^) were not affected by dSi addition (Table 2).

**TABLE 2:** Average growth rates of the Kalmar Algae Collection (KAC) picoeukaryote strains (KAC 117, KAC 118 and KAC 119) and *M. commoda* strain RCC827 and *O. tauri* strain RCC4221 cultured in control conditions (no added dSi) versus dSi‐enriched media (+100 μM)

Strain	Growth rate mean (day^−1^) (±SD)						Independent *t*‐test (*t*, df)		
	E1 control	E1 dSi‐enriched	E2 control	E2 dSi‐enriched	E3 control	E3 dSi‐enriched	E1	E2	E3
KAC 117	1.12 (±0.04)	1.13 (±0.04)	1.08 (±0.02)	1.12 (±0.02)	1.21 (±0.09)	1.06 (±0.07)	0.90 (0.13, 4)	0.32 (1.14, 4)	0.37 (1, 4)
KAC 118	1.01 (±0.02)	0.93 (±0.06)	1.60 (±0.01)	1.49 (±0.01)	1.63 (±0.04)	1.61 (±0.03)	0.43 (0.87, 4)	0.54 (0.68, 3)	0.79 (0.28, 4)
KAC 119	1.04 (±0.04)	1.12 (±0.04)	1.83 (±0.02)	1.75 (±0.01)	1.49 (±0.03)	1.36 (±0.06)	0.33 (1.1, 4)	0.09 (2.27, 4)	0.18 (1.6, 4)
RCC827	1.09 (±0.05)	1.25 (±0.03)	–	–	–	–	0.12 (1.94, 4)	–	–
RCC4221	1.36 (±0.05)	1.36 (±0.09)	–	–	–	–	0.98 (0.03, 4)	–	–

*Note*: Independent *t*‐test results are shown comparing the growth rates of the control and dSi‐enriched cultures for each cell line.

## DISCUSSION

In this study we demonstrated that two species of picoeukaryotes, belonging to different genera in the order Mamiellales and three novel Baltic Sea isolates from the order Trebouxiophyceae, accumulate bSi. All experiments signalled consistent bSi accumulation across isolates from both brackish and marine environments. The average amount of bSi accumulated by the studied picoeukaryotes in media amended with 100 μM dSi (ranging from 30 to 92 amol Si cell^−1^) is comparable to the average amount accumulated by *Synechococcus* strains cultured in dSi‐enriched (120 μM dSi) media (all clones <50 amol Si cell^−1^; Brzezinski et al., 2017). In control conditions (i.e. media with naturally occurring 0.70–2.99 μM dSi), the studied picoeukaryotes accumulated significantly lower amounts of bSi. Biosilicification has not previously been observed in laboratory cultures of these picoeukaryote classes which, like *Synechococcus*, do not seem to have any known silicon requirement for growth or metabolism.

Culturing in dSi‐enriched media had no observable effect on growth rates of the tested picoeukaryotes, suggesting that organisms from these picoeukaryotic groups lack an obligate need for silicon. This lack of an effect of dSi‐enriched media on the growth rates of the cultures was similarly noted in experiments with *Synechococcus*, most of which also do not have known SIT/SIT‐L genes (Brzezinski et al., 2017; Tostevin et al., 2021). Similarly, publicly available genome sequences on GenBank from *Ostreococcus* and *Micromonas* do not have annotated SIT/SIT‐L genes, and the mechanism(s) behind the bSi accumulation remains unknown. Notably, cells did not accumulate toxic amounts of bSi, as evidenced by the similar growth curves of control and dSi‐enriched cultures (Figure S2). Moreover, the total amount of bSi accumulation in the dSi‐enriched cultures, based on the cell abundances calculated at the final timepoint, was a small fraction of the amount of dSi added to the media (Table S4). Brzezinski et al., 2017 linked slower growth with higher bSi accumulation in *Synechococcus* cultures, which suggests that the accumulation results in our study may be conservative. Unlike the picoeukaryotes in our study, some picoeukaryotes have an identified biological need for silicon. For example, *Triparma laevis* (Bolidophyceae), which has some picoeukaryotic‐sized cells that measure <3 μm in diameter, uses dSi to construct silica shields around individual cells (Booth & Marchant, 1987; Kuwata et al., 2018; Yamada et al., 2014). *T. laevis* has some cellular structures for formation of silica shields that are analogous to diatoms but, unlike most diatoms, seems to lack an obligate need for dSi for growth (Yamada et al., 2016; Yamada et al., 2018). However, any potential purpose of bSi accumulation in most picophytoplankton is currently unknown.

Evaluating the effect of bSi accumulation in picoeukaryotes can affect our understanding of carbon cycling. Picoeukaryotes have lower estimated global cell abundances (1.6 × 10^26^ ± 0.2 × 10^26^ cells; Flombaum et al., 2020) compared with the picocyanobacteria *Synechococcus* (7.0 × 10^26^ ± 0.3 × 10^26^) and *Prochlorococcus* (2.9 × 10^27^ ± 0.1 × 10^27^; Flombaum et al., 2013). Despite this, picoeukaryotes can dominate carbon cycling (Goericke, 1998; Grob et al., 2007; Li, 1994), particularly in coastal waters where picoeukaryotes like *Ostreococcus* lead net carbon production and consumption (Worden et al., 2004). Coastal areas are also associated with higher concentrations of dSi, which is frequently replenished via upwelling (Nelson et al., 1981) or direct terrestrial runoff. At the coastal sampling site where the Baltic Sea strains in our study were isolated, photosynthetic picoeukaryotes were major contributors to the total phytoplankton biomass (up to 73%; Alegria Zufia et al., 2021). This significant contribution to total biomass calls for further investigation of bSi accumulation by picoeukaryotes to elucidate their potential role in silica cycling.

Field studies measuring bSi in the picoplankton size fraction have linked bSi variability to changing biological dynamics rather than environmental factors (Krause et al., 2017; Leblanc et al., 2018; Wei, Wang, et al., 2021). In a field study in the Eastern Indian Ocean, the amount of bSi accumulation in the pico‐sized phytoplankton fraction at different stations was influenced by biotic, rather than abiotic factors (Wei, Wang, et al., 2021). In fact, the correlation between bSi accumulation in the ≤2 μm fraction and the quantity of different pico‐sized groups (*Synechococcus*, *Prochlorococcus*, picoeukaryotes) was only significant for picoeukaryotes (Wei, Wang, et al., 2021). Though this was a ‘local’ level correlation observed in the Eastern Indian Ocean, the effect on global marine bSi production should be investigated. Accumulation of bSi can also inadvertently change the physical properties of the cells. For example, it was recently hypothesized that possible bSi accumulation may promote sinking of picophytoplankton cells and thereby promote carbon fluxes into sediment (Richardson, 2019). Though it is unknown if bSi accumulation is under selective pressure in picoeukaryotes, this can have important environmental consequences.

Culture conditions and the methods used for measuring bSi may influence the results in studies of bSi accumulation. The experimental set‐up and analyses used in this study were designed specifically to have comparative results to the studies of bSi accumulation in *Synechococcus*, using 100 μM dSi‐enriched media for incubations and NaOH digestion and the silicomolybdate method for analysis of dSi and bSi concentrations in the media and cells, respectively (Baines et al., 2012; Brzezinski et al., 2017). We closely monitored the pH of the cultures to prevent the formation of the mineral sepiolite, which can form at pH >8.5 and compromises cellular silicon measurements (Nelson et al., 1984). Similar to what has been noted in *Synechococcus* strains (Baines et al., 2012, Brzezinski et al., 2017), the picoeukaryote cultures exhibited variability in the amount of bSi accumulated between the biological triplicates and between the experiments. In the field, bSi varied in individual *Synechococcus* cells collected from the same environment (Ohnemus et al., 2016), and the differences in average bSi accumulation within different experiments using the same cell line indicate that this variation can be present in laboratory cultures. The reason for this variability will likely remain unexplained until the cellular location, structural form, and physiological role of silicon is determined in *Synechococcus*, Mamiellales and Trebouxiophyceae cells. Ohnemus et al. (2018) reported that *Synechococcus* cells contain a spectrally distinct, more‐ordered form of bSi that is different from the amorphous biogenic opal that makes up diatom frustules. They compared the typical NaOH digestion for a 2.5 M hydrofluoric‐acid digestion that yielded significantly higher amounts of bSi in the same sample. Thus, accumulation of dSi in the current study may have been underestimated, potentially implying that the ecological influence of biosilicification in picoeukaryotes on marine ecosystems is yet larger than currently recognized.

Our study focused on five picoeukaryote strains, but our results hold broader implications for knowledge about the generation of bSi in the oceans. This study confirms that biosilicification is widespread, both in ubiquitous and well‐characterized marine species and in novel brackish picoeukaryote strains. bSi accumulation in the tested picoeukaryote strains proceeds via a yet unknown mechanism but appears to be similar to uptake mechanisms in *Synechococcus*. The most recent revision of the global silicon budget (Tréguer et al., 2021) included a discussion about bSi production and accumulation in the pico‐sized fraction though, as the most thus far studied group of biosilificiers in this fraction, the focus was primarily on *Synechococcus*. We recommend that future field studies of bSi in the pico‐sized fraction should consider the contribution of picoeukaryotes, especially in coastal areas, and that picoeukaryotes should be included as potential biological sources of bSi in future silica budgets.

## AUTHOR CONTRIBUTIONS

**Yelena Churakova:** Formal analysis (lead); investigation (lead); visualization (lead); writing – original draft (lead). **Anabella Aguilera:** Methodology (equal); writing – original draft (supporting); writing – review and editing (equal). **Evangelia Charalampous:** Methodology (equal); writing – review and editing (equal). **Daniel J. Conley:** Conceptualization (equal); funding acquisition (equal); writing – review and editing (equal). **Daniel Lundin:** Conceptualization (equal); data curation (lead); funding acquisition (equal); supervision (supporting); writing – review and editing (equal). **Jarone Pinhassi:** Conceptualization (equal); funding acquisition (equal); supervision (supporting); writing – review and editing (equal). **Hanna Farnelid:** Conceptualization (equal); funding acquisition (equal); supervision (lead); writing – original draft (supporting); writing – review and editing (equal).

## CONFLICT OF INTEREST

The authors declare no conflict of interest.

## Supporting information


**DATA S1.** Supporting Information
