# Supplementary material for: Biogenic silica accumulation in picoeukaryotes: Novel players in the marine silica cycle
Source: Environ Microbiol Rep. 2023 Mar 29;15(4):282–90. doi: 10.1111/1758-2229.13144 (PMC10316375; doi:10.1111/1758-2229.13144)

**Supporting Information:**

**Table S1**. Average bSi accumulation per cell in individual biological replicates for all cell lines and experiments conducted. Biological triplicate cultures were used in most experiments, except KAC 118 in E2, which used biological duplicate cultures.

| **Experiment** | **Cell Line** | **Control (amol Si cell^-1^)** | **dSi-enriched (amol Si cell^-1^)** |
| --- | --- | --- | --- |
| E1 | KAC 117 | 19.68, 23.55, 28.73 | 66.12, 48.90, 65.41 |
|  | KAC 118 | 12.72, 12.70, 7.50 | 27.58, 54.60, 42.51 |
|  | KAC 119 | 6.68, 11.12, 10.27 | 18.87, 22.88, 21.38 |
|  | RCC827 | 33.24, 33.46, 29.77 | 57.30, 56.91, 59.89 |
|  | RCC4221 | 43.17, 43.76, 48.22 | 74.44, 82.93, 119.85 |
| E2 | KAC 117 | 31.54, 39.76, 34.01 | 59.97, 70.72, 95.80 |
|  | KAC 118 | 20.19, 28.24 | 61.85, 61.75, 57.49 |
|  | KAC 119 | 15.25, 7.30, 11.19 | 46.61, 47.78, 37.14 |
| E3 | KAC 117 | 31.25, 29.96, 39.37 | 38.39, 54.24, 59.29 |
|  | KAC 118 | 16.96, 16.47, 14.63 | 35.10, 26.72, 34.38 |
|  | KAC 119 | 11.13, 12.58, 23.4 | 24.03, 22.63, 31.44 |

**Table S2.** T-test comparing average bSi accumulation in control and dSi-enriched conditions for each cell line and during E1, E2, and E3. Significant (<0.05) values are in bold.

| **Cell Line** | **Independent t-Test (t, df)** |  |  |
| --- | --- | --- | --- |
|  | **E1** | **E2** | **E3** |
| KAC 117 | **<0.01** (5.83, 4) | **0.02** (3.71, 4) | 0.07 (2.46, 4) |
| KAC 118 | **0.02** (3.82, 4) | **<0.01** (10.24, 3) | **<0.01** (5.78, 4) |
| KAC 119 | **<0.01** (6.52, 4) | **<0.01** (7.99, 4) | 0.09 (2.18, 4) |
| RCC827 | **<0.0001** (17.07, 4) | **–** | **–** |
| RCC4221 | **0.03** (3.38, 4) | **–** | **–** |

**Table S3.** Average biovolume (N = 20 – 30 cells) and average bSi accumulation (from E1, E2, and E3) of each cell line.

| **Cell Line** | **Average biovolume µm^3^ (± SD)** | **Average bSi amol Si cell^-1^ (± SD)** |
| --- | --- | --- |
| KAC 117 | 2.03 (±1.18) | 62.09 (±16) |
| KAC 118 | 1.26 (±0.50) | 44.66 (±14.43) |
| KAC 119 | 1.29 (±0.61) | 30.31 (±11.09) |
| RCC827 | 3.99 (±1.21) | 58.04 (±1.62) |
| RCC4221 | 1.32 (±0.40) | 92.41 (±24.14) |

**Table S4.** Average dSi of triplicate cultures in the media measured at the final timepoint of each experiment in both control and dSi-enriched media conditions. Total accumulation of Si was calculated in dSi-enriched cultures, using average bSi accumulation measurements.

| **Experiment** | **Cell Line** | **Control (µM ± SD)** | **dSi-enriched µM (± SD)** | **Total Accumulation Si µM (± SD)** |
| --- | --- | --- | --- | --- |
| E1 | KAC 117 | 0.62 (±0.22) | 88.87 (±2.05) | 0.40 (±0.04) |
|  | KAC 118 | 0.56 (±0.22) | 88.76 (±4.39) | 0.43 (±0.03) |
|  | KAC 119 | 0.54 (±0.18) | 88.76 (±1.99) | 0.34 (±0.03) |
|  | RCC827 | 3.43 (±0.54) | 96.96 (±3.15) | 0.47 (±0.02) |
|  | RCC4221 | 2.29 (±0.38) | 103.70 (±17.27) | 0.58 (±0.04) |
| E2 | KAC 117 | 0.76 (±0.27) | 97.29 (±2.34) | 0.25 (±0.04) |
|  | KAC 118 | 1.07 (±0.55) | 97.25 (±3.97) | 0.72 (±0.03) |
|  | KAC 119 | -0.03 (±0.06) | 92.18 (±4.30) | 0.58 (±0.1) |
| E3 | KAC 117 | 0.83 (±0.05) | 98.49 (±6.91) | 0.16 (±0.07) |
|  | KAC 118 | 1.19 (±0.33) | 106 (±13.73) | 0.25 (±0.01) |
|  | KAC 119 | 1.10 (±0.21) | 96.95 (±9.38) | 0.18 (±0.04) |

**Figure S1.** Seasonal variation of ASVs identical to KAC isolates from Linnaeus Microbial Observatory 16S rRNA gene amplicon libraries spanning 2011-2020. Each point represents relative abundance (%) of KAC 117 associated and KAC 118, KAC 119 associated ASV at a specific sampling date. The figure was made using the ggplot2 package in R.


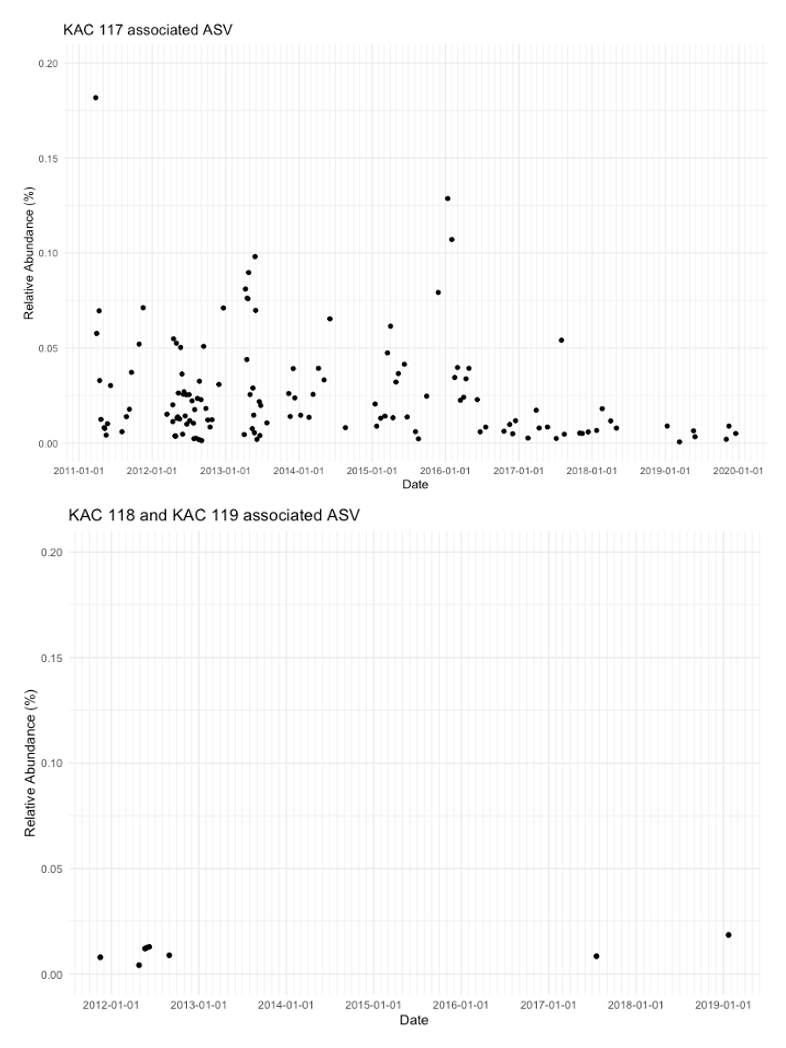


**Figure S2.** Growth curves of KAC isolates and marine strains during experiments E1-E3 (KAC 117, KAC 118, KAC 119) and E1 (RCC827, RCC 4221). The two curves on each graph represent cultures grown in control conditions (purple squares) and in dSi-enriched conditions (black triangles). Each curve is the average optical density (OD) of triplicate cultures, except KAC 118 E2 which is the average OD of duplicate cultures. Error bars represent standard deviation.


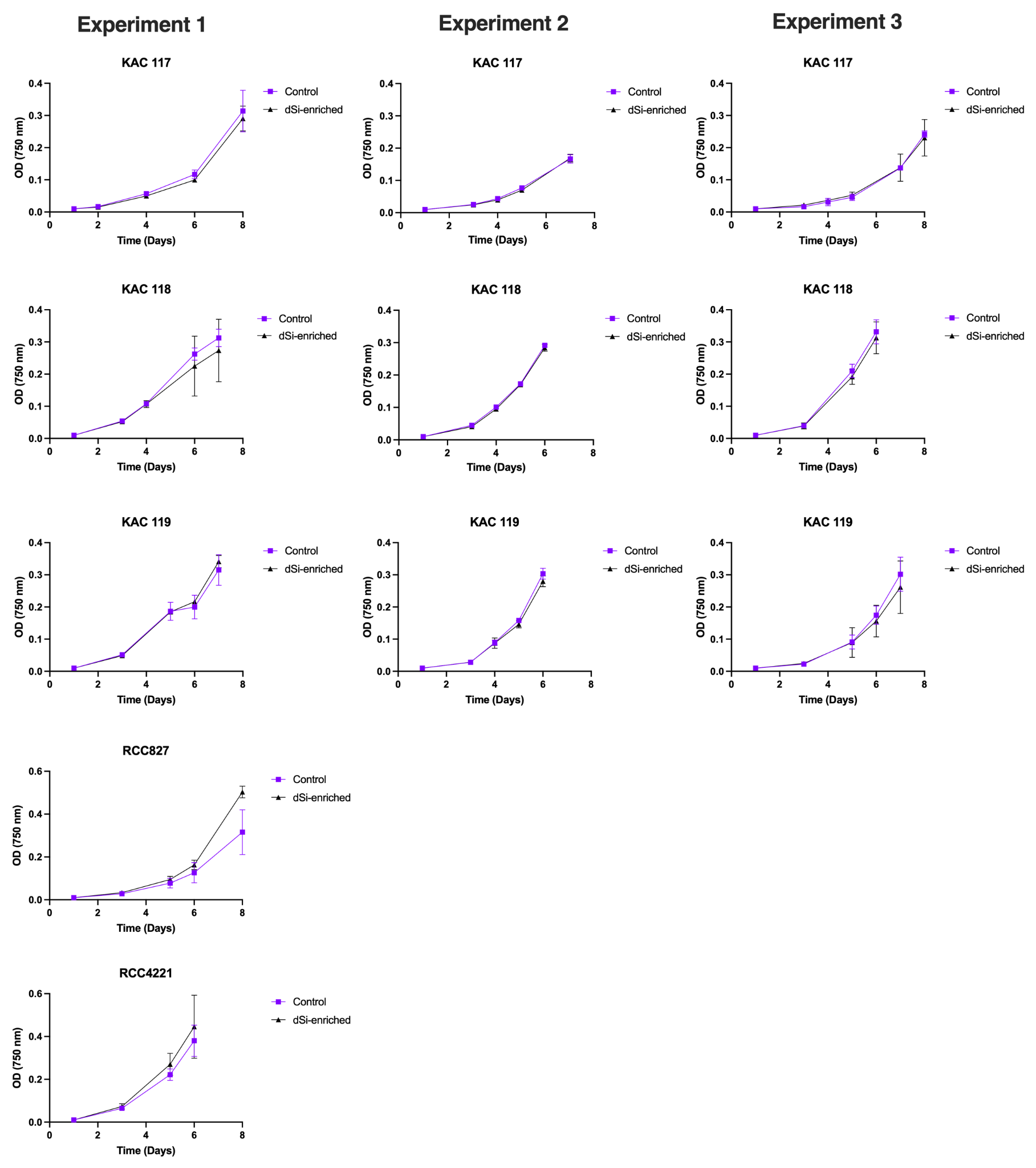


**Figure S3.** pH of KAC isolates and marine strains during experiments E1-E3 (KAC 117, KAC 118, KAC 119) and E1 (RCC827, RCC 4221). Similar pH trends were observed in most cultures during experiments. Outlier cultures are labeled on the graphs next to the endpoint of their pH curve. Control triplicate cultures are labeled A1, A2, A3 and dSi-enriched triplicate cultures are B1, B2, and B3.


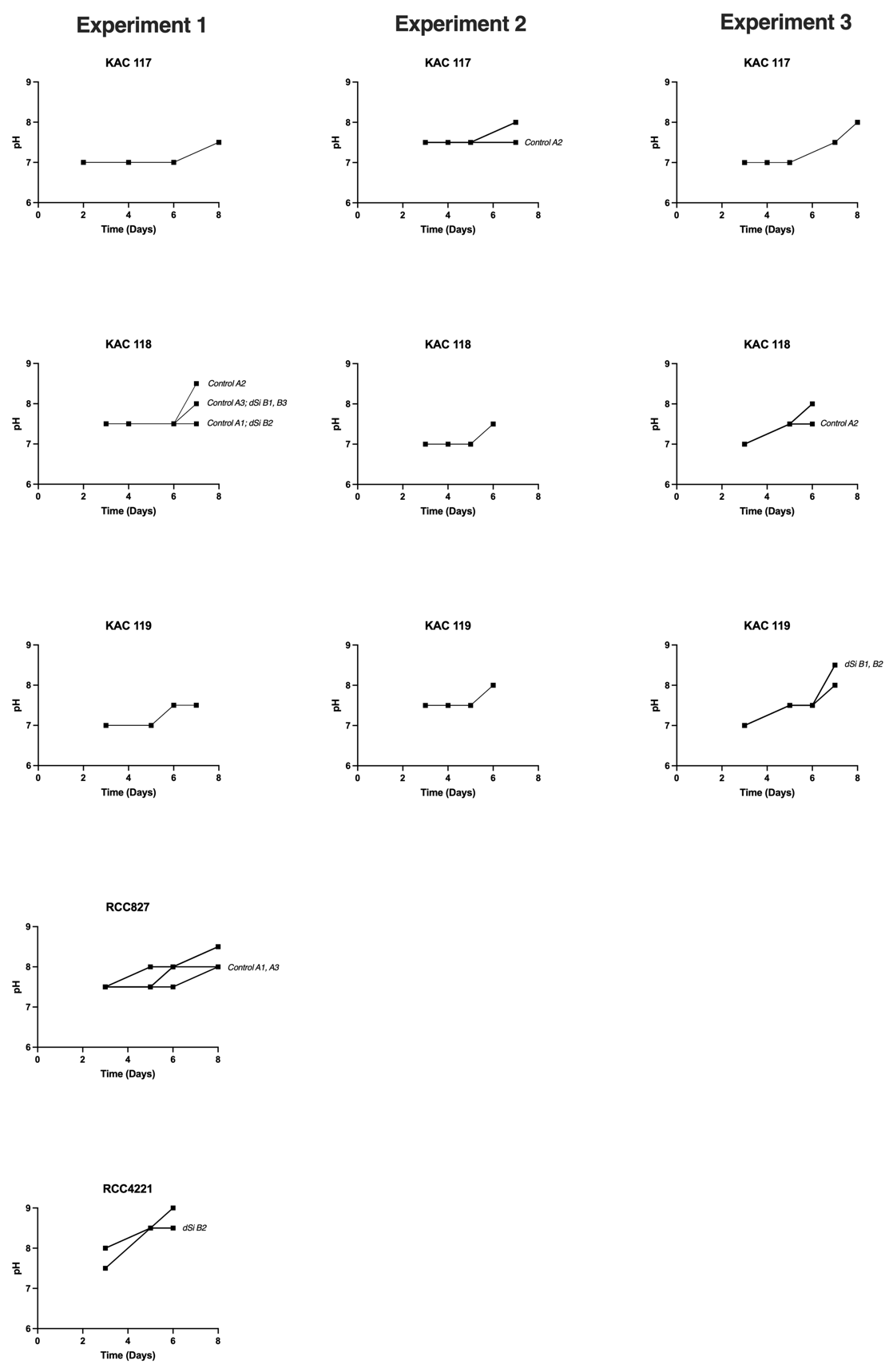

Supplement: Supplementary file 1 — DATA S1. Supporting Information [file EMI4-15-282-s001.docx]
